# Preoperative high-precision three-dimensional reconstruction in laparoscopic splenectomy for supramassive splenomegaly: a case report and literature review

**DOI:** 10.3389/fmed.2025.1570335

**Published:** 2025-03-05

**Authors:** Cheng Huang, Zhichao Gao, Yuhang Zhang, Lida Ge

**Affiliations:** ^1^Department of Colorectal Surgery, First People’s Hospital of Xiaoshan District, Hangzhou, Zhejiang, China; ^2^Department of Neurosurgery, First People’s Hospital of Xiaoshan District, Hangzhou, Zhejiang, China; ^3^Department of Orthopedics, First People’s Hospital of Xiaoshan District, Hangzhou, Zhejiang, China; ^4^Department of Hepatobiliary Surgery, First People’s Hospital of Xiaoshan District, Hangzhou, Zhejiang, China

**Keywords:** laparoscopic splenectomy, supramassive splenomegaly, three-dimensional reconstruction, preoperative planning, autoimmune hepatitis

## Abstract

**Background:**

Massive splenomegaly is considered to pose a high risk for laparoscopic splenectomy (LS). We report a case of supramassive splenomegaly wherein the patient successfully underwent LS guided by preoperative three-dimensional (3D) reconstruction.

**Case presentation:**

A 35-year-old female had a history of autoimmune hepatitis spanning 4 years, accompanied by progressive splenomegaly. Her spleen had grown to a size of 27.3 cm in diameter, and a consistent decline in her blood cell counts had been noted over the same period. Considering the significant enlargement of the spleen and the technical challenges associated with LS in such instances, a preoperative 3D reconstruction was performed. This 3D model accurately delineated the splenic artery and depicted the positional relationships between the enlarged spleen and nearby organs, thus supporting detailed preoperative planning. Following the surgical route determined in the preoperative planning, 3D assistance enabled the safe ligation of the splenic artery and meticulous separation of the spleen from adjacent tissues. The patient’s postoperative recovery was smooth and free from complications.

**Conclusion:**

Meticulous preoperative 3D planning may help overcome technical difficulties and enable successful LS even in patients with supramassive splenomegaly.

## 1 Introduction

Splenectomy is frequently conducted through minimally invasive techniques, including laparoscopy, hand-assisted laparoscopy, and robotic surgery (1). Although laparoscopic splenectomy (LS) has been the standard technique for elective splenectomy since its initial description in 1991, dealing with massive splenomegaly presents notable technical challenges (2). While European guidelines recommend LS as the standard of care, its application in massive splenomegaly remains debated owing to the higher reported risks (3). Despite its widespread use, LS continues to present significant technical challenges in patients with massive splenomegaly. Massive splenomegaly was traditionally regarded as a relative contraindication to LS due to the enlarged spleen, complex anatomical structures, and heightened risks of intraoperative hemorrhage and conversion to open surgery (4). However, with advancements in luminal techniques and the accumulation of surgical experience, the safety and efficacy of LS in the treatment of giant spleens have gained increasing recognition (5). Further improvements in surgical skills and the development of advanced equipment have also played a pivotal role in expanding the feasibility of LS for massive splenomegaly (6). Notably, preoperative planning aided by three-dimensional (3D) reconstruction technology allows for a more precise evaluation of the anatomical relationships between abdominal organs and surrounding tissues, thereby enhancing the safety and success rates of surgery (7).

We aimed to evaluate the feasibility of a minimally invasive approach when using preoperative planning aided by 3D reconstruction technology in a case with massive splenomegaly.

## 2 Case description

### 2.1 Patient information

**Figure 1:**
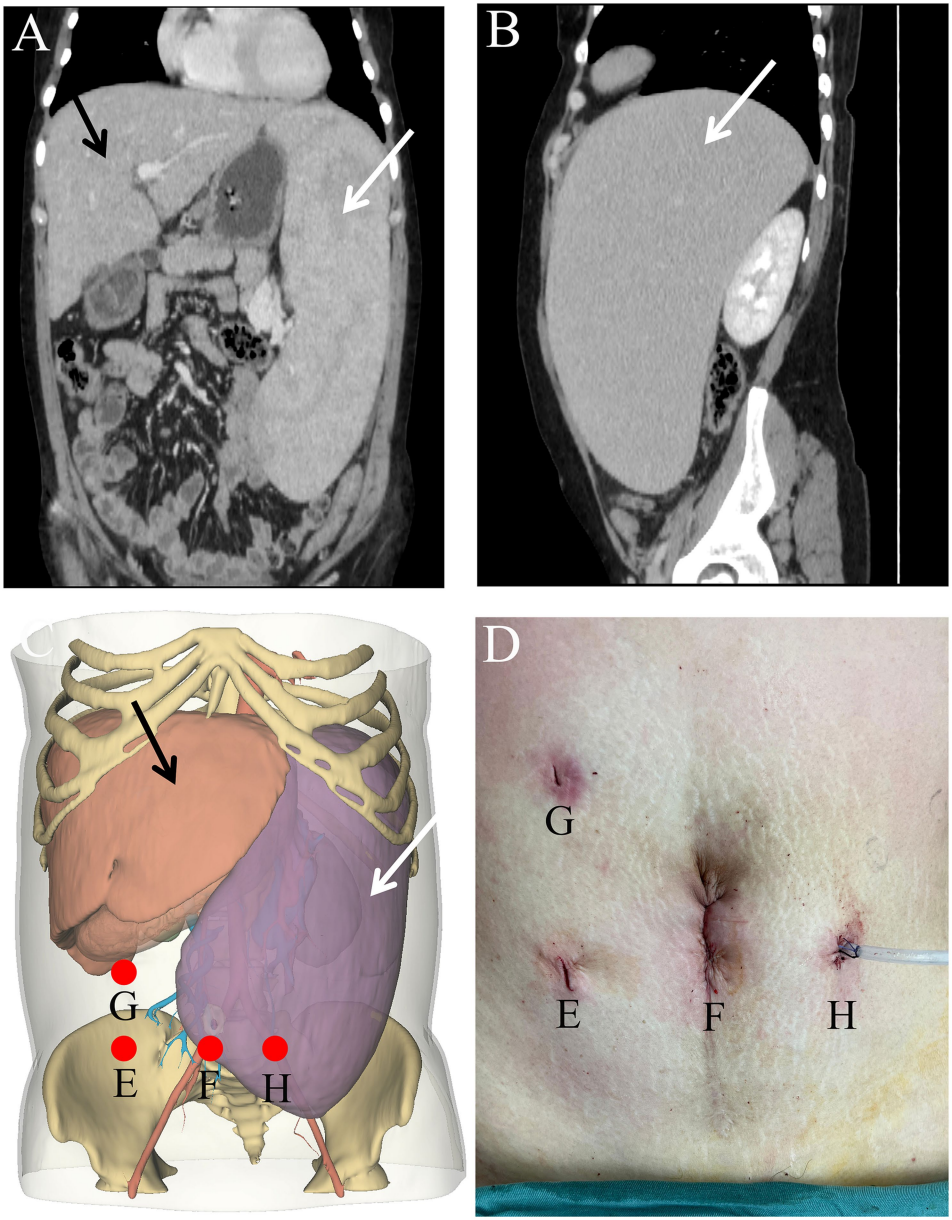
Coronal **(A)** and axial **(B)** views of the computed tomography images showing the spleen measures 27.3 cm in length, 6.0 cm in thickness, and 19.4 cm in width. Comparison of preoperatively planned trocar positions **(C)** and actual surgical sites **(D)**. Location of surgical incision **(E–H)**. The white arrow indicates the splenic artery, while the black markings denote the splenic vein.

A 35-year-old female presented with autoimmune hepatitis (AIH) and a significantly enlarged spleen. Over the preceding 4 years, she had experienced a gradual decrease in blood cell counts. Physical examination demonstrated significant enlargement of both the liver and spleen, with the spleen extending beyond the midline and its lower boundary descending into the pelvis. The spleen exhibited a firm consistency and smooth surface without tenderness. Mild pitting edema was noted in both lower extremities, with a 2-mm indentation lasting 3–5 s upon finger pressure. Laboratory investigations revealed red and white blood cell and platelet counts of 2.5 × 10^9^/L (3.80–5.10 × 10^9^/L), 1.94 × 10^9^/L (3.50–9.50 × 10^9^/L), and 47 × 10^9^/L (125–350 × 10^9^/L), respectively. Hemoglobin level was 85 g/L (115–150 g/L) and the platelet hematocrit was 0.036% (0.110–0.280%). Liver function tests revealed elevated serum aminotransferases as follows: alanine transaminase 4 U/L (7–40 U/L), aspartate transaminase 43 U/L (13–35 U/L), gamma-glutamyl transferase 175 U/L (7–45 U/L), and alkaline phosphatase 151 U/L (35–100 U/L). Immunoglobulin (Ig) levels were as follows: IgE 3 kIU/L (0–100 kIU/L), IgG 22.28 g/L (7.00–16.00 g/L), IgM 6.10 g/L (0.40–2.30 g/L), and IgA 2.61 g/L (0.70–4.00 g/L). Liver autoimmune antibody tests showed positivity for antinuclear antibodies, strong positivity for anti-gp210 antibodies, positivity for anti-mitochondrial antibodies, and moderate positivity for anti-mitochondrial M2 antibodies. Abdominal computed tomography (CT) revealed hepatosplenomegaly, with the spleen measuring 27.3 cm in maximum length, 6.0 cm in thickness, and 19.4 cm in width, with the splenic vein dilated to 21 mm at the splenic hilum (Figures 1A,B). In summary, the patient was ultimately diagnosed with autoimmune hepatitis, portal hypertension, and splenomegaly accompanied by hypersplenism.

### 2.2 3D reconstruction and preoperative planning

#### 2.2.1 CT scanning parameters

The patient underwent a contrast-enhanced CT scan. The scan was performed with a slice thickness of 1.25 mm. The contrast agent used was iohexol, and it was injected at a rate of 3–4 mL/s with a total dose of 100 mL, which was adjusted according to the patient’s body weight and renal function. A volumetric acquisition protocol was optimized using a slice thickening of 1.25 mm [2.5 mm reconstructions; CT Equipment: Philips Ingenuity 128 Slices, Royal Philips (RP), Amsterdam, Netherlands]. The radiation dose length product was 50 mGy/cm with an estimated effective dose to the patient of about 0.3 mSv.

#### 2.2.2 Model registration method

We used the open-source software 3D Slicer in two different approaches to generate 3D models from abdominal CT. The first approach involves using density differences to differentiate various solid organs. In this model, the threshold range for each organ was as follows: spleen (59.00–133.00), liver (81.00–118.00), and pancreas (58.00–98.00). Additionally, an alignment method based on multipoint landmarks was employed. First, several anatomical landmarks, such as the inferior vena cava and aorta, were identified on the two-dimensional (2D) CT images and the initially generated 3D model. The software then automatically calculated a transformation matrix to accurately align the 2D images within 3D space, ensuring the geometric precision of the model. Ultimately, both approaches require a professional to refine and adjust the model based on the CT images to achieve a high level of accuracy.

#### 2.2.3 Emergency plan

In the event of a massive hemorrhage during the operation, a well-defined emergency plan was in place to ensure patient safety. The criteria for conversion to open surgery were as follows: (1) Excessive blood loss: When the estimated blood loss exceeded 1,000 mL and could not be effectively controlled using laparoscopic techniques, such as endoscopic suturing or clip application. (2) Hemodynamic instability: If the patient exhibited signs of hemodynamic instability, including sustained hypotension (systolic blood pressure < 90 mmHg) and tachycardia (heart rate > 120 beats per minute), necessitating rapid blood transfusion. (3) Severely impaired visualization: When excessive bleeding completely obscures the laparoscopic field of view, making it impossible to identify anatomical structures. (4) Splenic hilar vessel injury: In cases of damage to the main trunk of the splenic artery or splenic vein that could not be adequately managed laparoscopically. During conversion to laparotomy, an experienced surgical team was prepared to achieve hemostasis rapidly. This included techniques such as ligation of major bleeding vessels or packing of the hemorrhagic site. Additionally, in cases where massive hemorrhage severely impaired anatomical structure identification, the preoperative 3D model was used as a reference to guide the surgery effectively.

#### 2.2.4 Preoperative planning and model matching

The longest diameter and volume of the spleen were measured, and the 3D reconstruction model of each organ was manipulated to enhance visualization, including combining, splitting, rotating, and adjusting transparency. The anatomical relationships were carefully observed, including the liver-spleen interaction, the position of the splenic artery outside the pancreatic parenchyma along the upper border of the pancreas, the spatial relationship between the splenic tail of the pancreas and the spleen, as well as the morphology of the splenic pedicle and its branches. The puncture site was planned based on this analysis, with the model projected onto the patient’s body surface using Persp 3D software (Med Insight Technology). The puncture site was marked on the patient’s abdomen, and ultrasound was used preoperatively to confirm the alignment of the spleen within the abdominal cavity.

### 2.3 Surgical procedure

**Figure 2:**
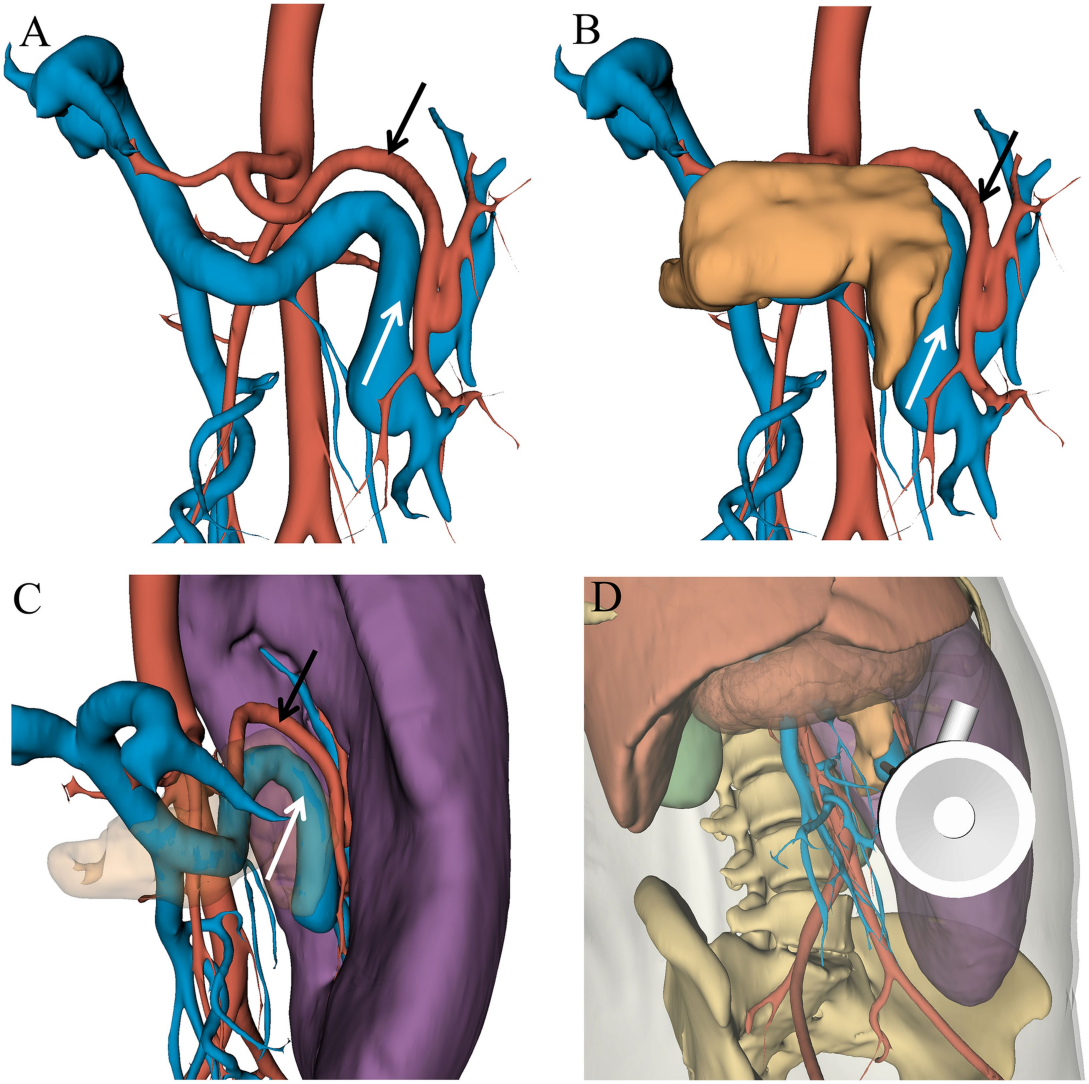
**(A)** Spatial relationship between the splenic artery and vein. **(B)** Spatial relationship between the pancreas and splenic artery. **(C)** Spatial relationship between the splenic artery and vein at the splenic hilum. **(D)** Simulated surgical view from the observation port (Figure 1E). The white arrow indicates the splenic artery, while the black markings denote the splenic vein.

**Figure 3:**
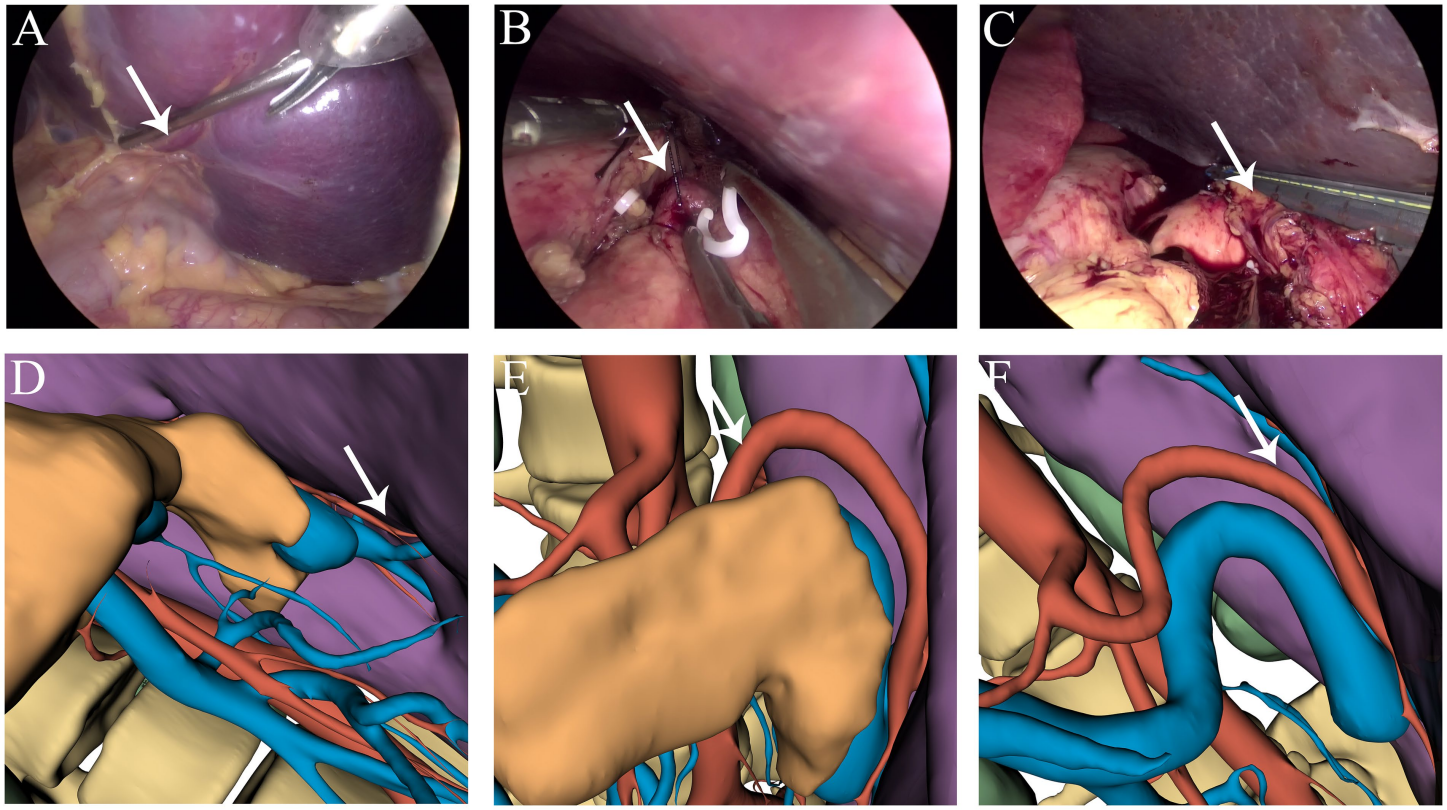
**(A,D)** Branches of the splenic artery. **(B,E)** Ligation of the splenic artery. **(C,F)** Dissection at the splenic hilum. The white arrow indicates the splenic artery.

Considering that the massively enlarged spleen occupied most of the abdominal cavity, open surgery was not ideal. Therefore, the feasibility of performing LS was assessed. Open-source software (3Dslicer software) was employed to create a 3D model of the abdomen from 2D CT images. This enabled us to identify the optimal trocar placement, elucidate the anatomy of the arteries and veins, and clarify the spatial connections between the enlarged spleen and neighboring intra-abdominal organs. Preoperative surgical planning using this 3D reconstruction identified feasible laparoscopic access points and enhanced the visualization of critical structures, thereby enabling the assessment of the technical feasibility of employing a minimally invasive approach in this special case (Figures 1C,D). Carbon dioxide was used to establish pneumoperitoneum at a pressure of 12 mm Hg. A 10 mm trocar was positioned just inferior to the point where the right midclavicular line meets the horizontal line through the umbilicus, designated as the observation port (Figure 1E). Following the preoperative surgical plan, a 12 mm trocar was inserted at the umbilicus, serving as the primary operating port (Figure 1F). A 5 mm trocar was placed at the meeting point of the right midclavicular line and horizontal umbilical line to serve as an assistant operating port (Figure 1G). An additional 5 mm trocar was placed just below where the left midclavicular line intersects with the horizontal line through the umbilicus for an assistant port (Figure 1H). Through the observation port, the course of the splenic artery branches was observed (Figure 2D). The splenic artery branches were dissected first before entering the lesser omental sac and exposing the upper margin of the pancreas using an ultrasonic scalpel. The splenic artery was ligated with an absorbable bio-clip at the upper edge of the pancreas (Figures 3A,B). Subsequently, the short gastric vessels were divided, and the gastrosplenic ligament was dissected and secured. The tissue at the splenic hilum was cut using a linear cutter and stapler (Figures 3C–F). After complete resection of the spleen, the spleen was completely freed from the surrounding structures and removed in portions, placed in a specimen retrieval bag, crushed, and extracted through the umbilical lower puncture site (Figure 1F). A drainage tube was inserted through the assistant port (Figure 1H) to drain the splenic fossa. The total operating time was 135 min, with an estimated blood loss of 250 mL.

### 2.4 Postoperative course

**Figure 4:**
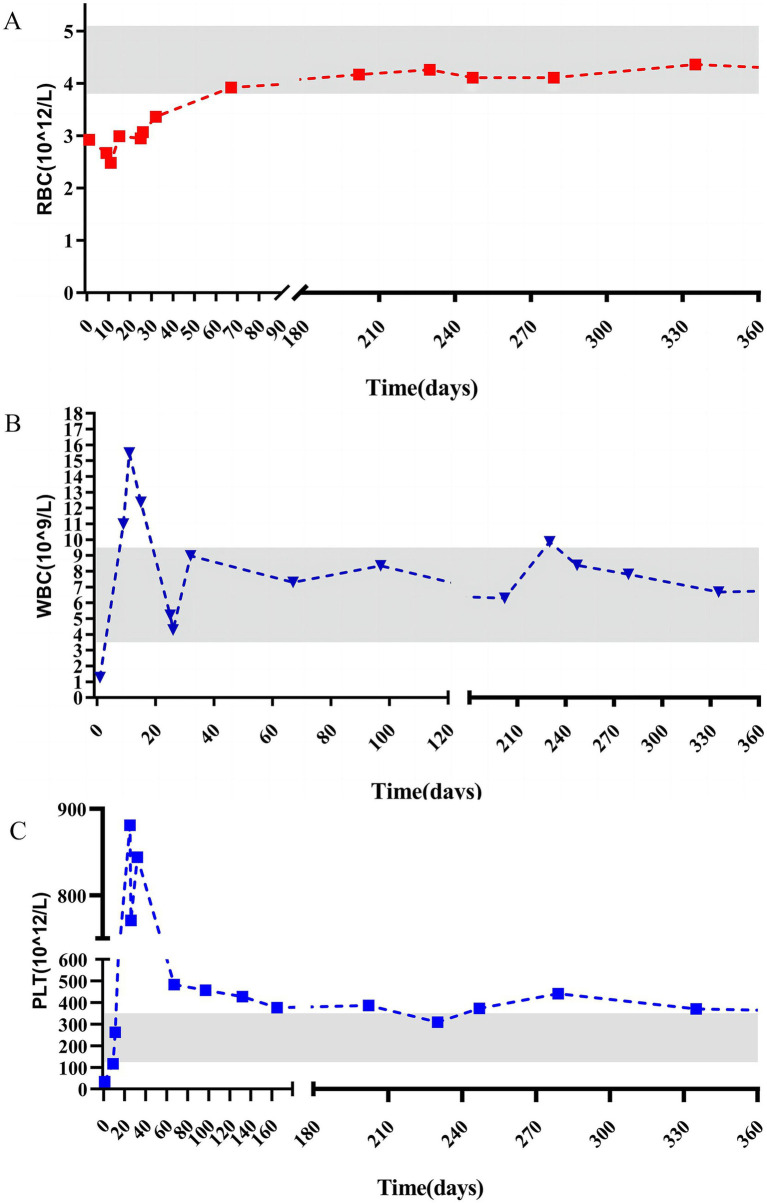
Pre and postoperative changes in red blood cell **(A)**, white blood cell **(B)**, and platelet **(C)**.

According to the Clavien–Dindo classification system, the patient experienced a Grade 1 postoperative complication. The drainage tube was removed on postoperative day 5, and the patient was discharged on day 7. Monthly follow-up appointments were arranged. Laboratory test results at 60 d postsurgery were within normal ranges. The patient was followed up for 16 months, during which all hematological indices have remained stable. These dynamic changes in platelet count (Figure 4) clearly demonstrate the efficacy of splenectomy in facilitating platelet recovery.

## 3 Discussion

### 3.1 Disease background and traditional surgical challenges

AIH is an inflammatory disease of unknown etiology that can progress to liver cirrhosis and end-stage liver failure if left undiagnosed and untreated (8). It can affect individuals of all ages and populations, regardless of race or ethnicity, with a reported worldwide annual incidence of 1.37 per 100,000 people and a prevalence of 17.44 per 100,000 (9). The pathophysiology of AIH involves an abnormal immune response targeting liver tissues, leading to ongoing inflammation that can progress to liver fibrosis, cirrhosis, and failure if not managed (10). Additionally, portal hypertension (a common complication of liver diseases, including AIH) can cause splenic congestion and the subsequent development of splenomegaly and increase the risk of death (11).

Massive splenomegaly is characterized by abnormal splenic enlargement, which can result in anemia, bleeding tendencies, and splenic hyperfunction (12). The classification of splenomegaly varies, with some authors defining spleens 17–22 cm in size as “massive” and those weighing >1,600 g or > 22 cm as “supramassive” (13). Splenectomy can effectively alleviate symptoms of massive splenomegaly and prevent complications, such as splenic infarction and hyperfunction (14). According to Smith et al.’s classification, the longitudinal axis of the spleen, in this case, measured 27.3 cm, categorizing it as supramassive, which poses a higher risk in laparoscopic surgery. Laparoscopy has been considered the standard approach for splenectomy when the organ is ≤1,300 g and < 22 cm in diameter, but it is safe and feasible for larger spleens, with consideration of the increased risk of conversion to open surgery (15). LS is one of the most commonly utilized modalities for splenectomy. This minimally invasive approach is associated with reduced intraoperative bleeding, a lower incidence of postoperative complications, and shorter postoperative recovery times (16). Laparoscopic surgery in patients with splenomegaly faces a higher risk of conversion to open splenectomy and greater morbidity risks than those associated with normal-sized spleens owing to challenges such as a limited working space and potential trauma during retraction of adjacent organs or veins (17). Recent evidence suggests that laparoscopy may be more effective than open surgery in cases of massive splenomegaly (15).

### 3.2 Comparison with traditional surgery

Additionally, LS has demonstrated significant advantages in managing complex splenic conditions, such as splenomegaly or splenic rupture (6, 18). However, in patients with giant splenomegaly, the procedure is often more challenging, with longer operative times, extended postoperative hospital stays, and an increased risk of conversion to open surgery. In cases of laparoscopic megasplenectomy or ultramegasplenectomy, the operating space becomes significantly restricted, with peripheral organs and blood vessels being displaced and deformed. This increases the risk of inadvertent injury to surrounding blood vessels during the procedure, potentially leading to accidental hemorrhage (19). Recent technological advancements have made soft tissue reconstruction of the abdominal cavity feasible, enabling the integration of 3D reconstruction and augmented reality laparoscopic navigation (ARLN) into abdominal surgery (20). 3D Slicer was used to reconstruct contrast-enhanced CT scans of the patient, successfully transforming 2D images into 3D models. Compared to traditional 3D reconstruction models, this advanced model offers enhanced accuracy in visualizing tissues and organs surrounding the spleen, including the liver, pancreas, stomach, gastrosplenic artery, portal vein, and skin (Supplementary Table S1) (21). The detailed 3D model vividly illustrates how the splenic artery is positioned relative to the pancreas, as well as the tortuous branches of the splenic artery near the splenic hilum, aiding in pinpointing the best ligation site (Figure 2). Moreover, we could accurately visualize the vascular anatomy of the splenic hilum by simulating the procedure in advance and marking the trocar insertion points in the abdomen (Figures 1C, 2D), offering significant benefits over traditional laparoscopic surgery. Although the advantages of precise abdominal 3D models are significant, team members need to undergo specialized training in the appropriate software and must practice extensively.

### 3.3 Mechanism of 3D reconstruction technology by 3D slicer

3D Slicer is one of the most versatile and widely used software tools for 3D reconstruction in the medical field, enabling the efficient conversion of DICOM files into 3D models in various formats (22). Before reconstruction, medical images must be processed into the DICOM format, which includes spatial location meta-information (23). Segmentation is typically performed using algorithms such as Threshold and GrowCut. Threshold segmentation operates by selecting regions based on specified gray value ranges, often requiring manual adjustment of threshold parameters during application (24). Conversely, GrowCut employs a competitive region-growing approach that uses initial seed points and iteratively generates regions of interest and segmentations based on weighted similarity scores, making it particularly suitable for structures with complex shapes (25). With advancements in technology, deep learning-based single-view reconstruction methods have emerged, enabling the generation of 3D models from single images (26). Additionally, PyRadiomics, a Python-based, flexible open-source platform capable of standalone use or integration with 3D Slicer, offers the ability to extract large volumes of lesion feature data from medical images, facilitating advanced radiomic analyses (27).

### 3.4 Limitations of this study

Although this case report demonstrates the successful application of preoperative 3D reconstruction and planning for LS in a case of massive splenomegaly, conclusions regarding the advantages and limitations of this approach are limited by the single-case design of this study. Rigorous investigations with larger sample sizes are needed to substantiate this case’s findings, delineate the outcomes better, and further evaluate the technique. This may provide robust evidence regarding the utility and potential benefits of 3D reconstruction-guided LS for massive splenomegaly. In cases where the patient requires emergency surgery, the operation should be performed promptly without spending time on 3D reconstruction, to avoid missing the optimal surgical window. In the future, advancements in artificial intelligence (AI) technology may enable the integration of AI programs such as ChatGPT or DeepSeek into 3D Slicer software, further enhancing the speed and accuracy of model construction. Furthermore, prospective studies or retrospective analyses are needed to evaluate the broader applicability of 3D-guided LS.

## 4 Conclusion

We present a case of massive splenomegaly that was successfully treated with laparoscopic total splenectomy. We accurately mapped the course of the splenic artery, clarified its spatial relationships with the surrounding structures, and established an optimal surgical pathway by utilizing preoperative 3D reconstruction technology. This approach significantly enhanced the precision and safety of the surgery. Thus, this case highlights the critical role of advanced imaging technology and meticulous surgical planning in ensuring the success of the procedure.

## Data Availability

The original contributions presented in the study are included in the article/Supplementary material, further inquiries can be directed to the corresponding author.
