# Supplementary material for: Preoperative high-precision three-dimensional reconstruction in laparoscopic splenectomy for supramassive splenomegaly: a case report and literature review
Source: Front Med (Lausanne). 2025 Mar 5;12:1570335. doi: 10.3389/fmed.2025.1570335 (PMC11920151; doi:10.3389/fmed.2025.1570335)
Supplement: Supplementary file 1 [file Table_1.doc]

| ****Comparison Items**** | ****3D Reconstruction-Assisted Surgery**** | ****Traditional Surgery**** |
| --- | --- | --- |
| ****Operation Time**** | In this case, it was 135 minutes. | The average operation time is approximately 170 minutes. |
| ****Blood Loss**** | In this case, intraoperative blood loss was 250 mL. | Average blood loss is 200-600 mL. |
| ****Complication Rate**** | No complications occurred during surgery. | Reported complication rates range from 15% to 20%. |
| ****Clarity of Surgical Field**** | The 3D model provides a detailed, multi-dimensional view of complex anatomical structures, improving localization and surgical precision. | The two-dimensional field of view offers a limited visualization of anatomical structures, requiring greater reliance on the surgeon’s experience. |
| ****Precision of Preoperative Planning**** | The 3D model facilitates accurate surgical pathway design, pre-surgical simulation, and risk assessment. | Relies on two-dimensional imaging and the surgeon's expertise. |
| ****Postoperative Recovery**** | Enhanced surgical precision and reduced trauma contribute to quicker postoperative recovery and shorter hospital stays. | Patients may experience a longer recovery time owing to increased intraoperative trauma and less precision in surgical techniques. |
| ****Patient Satisfaction**** | higher satisfaction due to fewer complications, shorter recovery times, and better overall surgical outcomes. | Lower patient satisfaction due to longer recovery periods or a higher risk of complications. |
| ****Learning Curve**** | Requires additional training to become proficient with the 3D reconstruction software and tools. | Relatively shorter learning curve as surgeons are typically more familiar with two-dimensional imaging and standard techniques. |
| ****Cost and Resource Utilization**** | 3D Slicer is free but requires the surgical team to master 3D reconstruction techniques.. | Lower initial costs due to the use of standard equipment and techniques but may incur higher overall costs due to longer operation times, higher complication rates, and extended hospital stays. |
| ****Surgeon Workload and Fatigue**** | Reduces cognitive workload during surgery by providing clearer anatomical details and aiding more precise planning. | Higher cognitive and physical workload due to reliance on two-dimensional imaging and manual interpretation of complex structures. |

**Supplementary Table 1 Comparison of Surgical Outcomes and Characteristics Between 3D Reconstruction-Assisted Surgery and Traditional Surgery**
